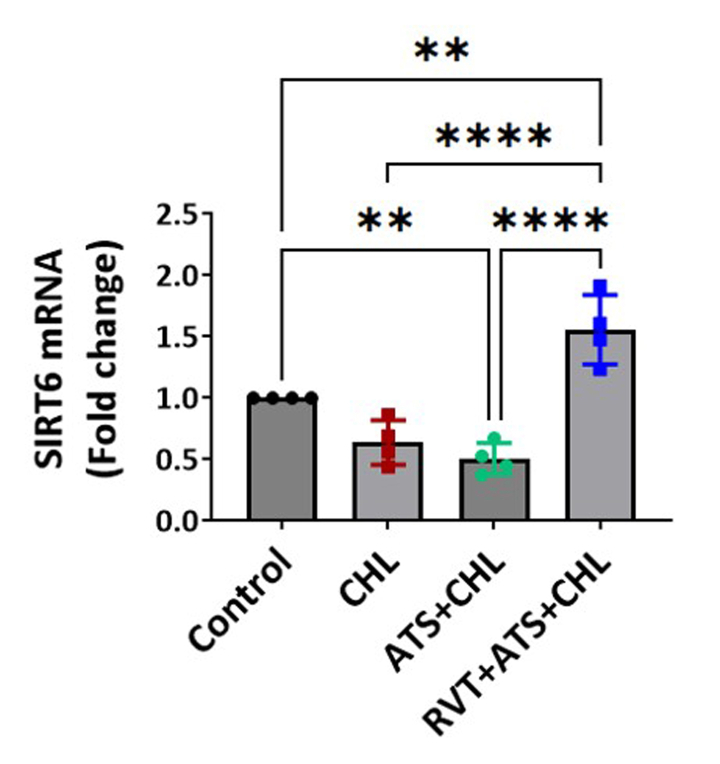# New epigenome players in the regulation of PCSK9-H3K4me3 and H3K9ac alterations by statin in hypercholesterolemia

**DOI:** 10.1016/j.jlr.2024.100699

**Published:** 2024-11-19

**Authors:** Sushmitha Duddu, Yash T. Katakia, Rituparna Chakrabarti, Pooja Sharma, Praphulla Chandra Shukla

**Affiliations:** 1 School of Medical Science and Technology, Indian Institute of Technology Kharagpur, Kharagpur, West Bengal, India; 2 Department of Biological Sciences, Birla Institute of Technology and Science (BITS), Pilani, India; 3 Department of Human Medicine, Carl von Ossietzky University, Oldenburg, Germany

**Keywords:** atorvastatin, cholesterol metabolism, epigenetics, histone modification, hypercholesterolemia

## Abstract

Statins are the most effective drugs used worldwide to lower the serum LDL-C by inhibiting the rate-limiting step, HMG-CoA reductase, in cholesterol biosynthesis. Despite its prevalent use, statins are known to increase proprotein convertase subtilisin/kexin type 9 (PCSK9) expression, hindering its efficiency. However, the underlying mechanisms remain elusive. In this study, we have unraveled the pleiotropic effects of statins on hypercholesterolemia via epigenetic regulation of PCSK9. We observed that atorvastatin (ATS) increases the fold enrichment of H3K4me3 at the promoter of PCSK9 by elevating the expression of the SET1/COMPASS family of proteins like SET1b and MLL1 in HepG2. In addition, ATS also acetylates H3K9 by increasing the expression of acetyltransferases like CBP and PCAF. Similarly, in mice fed a high-fat diet, ATS showed increased levels of H3K4me3 and H3K9ac in the liver. Furthermore, a pharmacological intervention that inhibits the H3K4me3 and H3K9ac enrichment resulted in the reversal of statin-induced upregulation of PCSK9. Combining statin and OICR-9429 or resveratrol improved the overall uptake of LDL by hepatocytes. Together, these findings suggest that statin induces the colocalization of H3K4me3 and H3K9ac to transcribe PCSK9 actively and that inhibiting these marks reduces PCSK9 expression and ultimately increases hepatocyte LDL uptake. Our study unveils a previously unknown epigenetic mechanism of PCSK9 regulation that may open new avenues to increase statin efficacy in patients and provide a potential therapeutic solution.

## Graphical abstract

**Figure:**
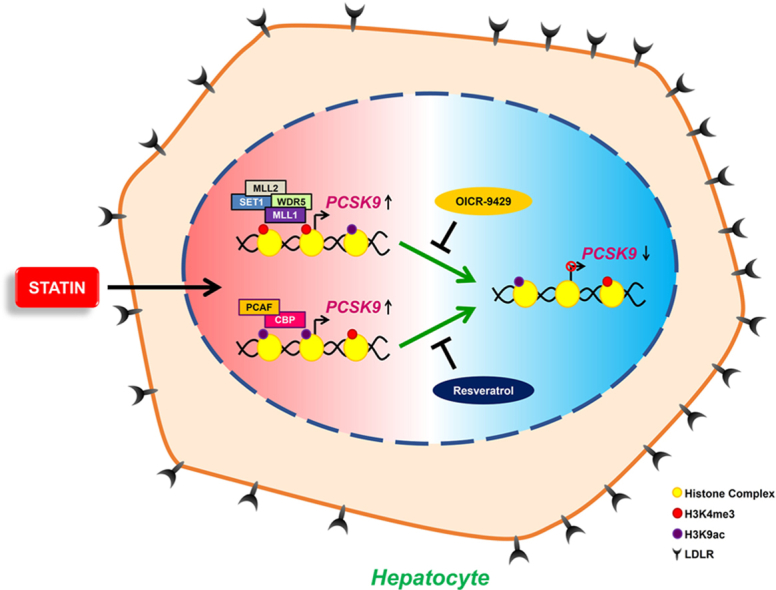

Statins are lipid-lowering drugs commonly used to reduce the risk of cardiovascular events by lowering circulating LDL-C. Statins are chemical inhibitors that competitively target the HMG-CoA reductase activity and block cholesterol biosynthesis in the liver (1). In the presence of high cholesterol, HMG-CoA reductase is subjected to feedback mechanisms via SREBP. SREBP is the key transcription regulator of cholesterol biosynthesis and the genes involved in the process. In addition to its inhibitory activity, statins are also widely recognized for altering the cholesterol-regulating gene by modulating the sterol-regulating element (SRE), which SREBP controls (2). Seidah *et al*. (3), for the first time, demonstrated that statin induces at least a 3-fold increase in the expression of proprotein convertase subtilisin/kexin type 9 (PCSK9) in hepatocytes. Similarly, many clinical studies have shown increased serum PCSK9 levels upon administration of statins in hypercholesterolemia patients (4). Studies indicate although the initial dosage of statins lowers the LDL-C, doubling the statin dosage further modestly lowers the LDL-C. Statins may counterproductively reduce the LDL-C because of increased PCSK9 and decreased LDL receptor (LDLR) (5, 6). However, the precise molecular mechanism responsible for the statin-mediated increase in the PCSK9 expression remains elusive, and understanding this would shed more light on statin intolerance and resistance in hypercholesterolemia patients.

PCSK9 is a recently discovered protein belonging to the proprotein convertase superfamily of serine proteinases. PCSK9 is an important LDL-C regulator. It binds and degrades LDLR on hepatocytes, thus rendering it unavailable for recycling and increasing serum LDL levels. Lately, PCSK9 inhibitors, mainly mAb like evolocumab and siRNA (inclisiran), are most effective in reducing LDL-C levels up to 50%–60% (7, 8). However, the long-term reduction in the PCSK9 levels requires more observations. Hence, it is crucial to understand the regulators of PCSK9 to lower the LDL-C in the patients effectively. Proximal transcription factors like SREBP2 bind to the SRE present in the proximal promoter region of *Pcsk9* to induce its expression in hepatocytes (9, 10, 11). In addition to SRE, the structural characterization of PCSK9 shows the presence of histone nuclear factor P, which acts as a coactivator that enhances the SREBP-mediated *Pcsk9* activation. The cofactors like NPAT (nuclear protein of the ataxia telangiectasia mutated locus) and TRRAP (transformation/transactivation domain-associated protein) form a functional complex and recruit histone acetyltransferase (HAT) to the promoter of Pcsk9 for active gene transcription (12). Sirtuins are another set of proteins known to regulate PCSK9 epigenetically. Previous studies have shown that FOXO3 recruits sirtuin 6 (sirt6) to the promoter region of *Pcsk9*, which further deacetylases histone 3 at lysine 9 and 56 positions, thus blocking the gene expression (13). A recent study revealed piceatannol reduces statin resistance by lowering PCSK9 levels via p300 acetyltransferase inhibition (14). However, few studies have reported the landscape of histone modifications at the PCSK9 gene promoter. Moreover, recent literature supports that statins regulate gene expression differently by modulating molecular and epigenetic pathways. Therefore, the present study was aimed at identifying the statin-induced histone modifications that epigenetically regulate the expression of PCSK9 in hepatocytes and demonstrates that statin epigenetically regulates PCSK9 by modulating the levels of H3K4me3 and H3K9ac in hepatocytes. It also increased the fold enrichment of these activating marks to the promoter region of *PCSK9*. Pharmacological inhibition of these histone marks reversed the statin-induced upregulation of PCSK9 and increased the uptake of LDL by the cells.

## Materials and methods

### Animals and treatment

Adult WT C57BL6 mice used in this study were housed in animal house with a facility providing individual ventilated cages with ad libitum access to food and water throughout the study. All the animals used in the study were male and aged between 6 and 8 weeks at the start of the high-fat diet (HFD). The animal house maintained a 12-12 h light and day cycle with an ambient temperature of 22–25°C with relative humidity controlled. All animal procedures used in this study were approved by the Institutional Animal Ethics Committee of IIT, KGP. A total of 16 animals were used for the study, and all were fed an HFD containing 1.25% cholesterol (D12108C; Research Diets, Inc, Haryana, India) for 20 weeks. The animals were randomly divided into two groups: one continued with HFD and the other with HFD + atorvastatin (ATS; 10 mg/kg). The statin was carefully measured and added to the animals' drinking water for 10 weeks. Animals were sacrificed, and the blood was collected by cardiac puncture for serum collection. The organs were harvested, immediately frozen using liquid nitrogen in Dewar flasks, and stored at −80°C until further molecular analysis.

### Cell culture

The HepG2 cells were purchased from the National Center of Cell Sciences, Pune, India. The cells were maintained at 37°C and 5% CO_2_ in complete media containing high glucose DMEM supplemented with 10% heat-inactivated (56°C for 30 min) fetal bovine serum (Gibco), 100 U/ml penicillin and streptomycin. For protein analysis, cells were seeded at a density of 0.3 × 10^6^ cells per well in 6-well plates in 1.5 ml of complete medium. On day 1, after 24 h of plating, the complete DMEM was replaced with Opti-MEM (Gibco, Thermoscientific, Mumbai, India) and incubated at 37°C; 5% CO_2_ for starvation. After the completion of starvation, the cells were incubated with DMEM containing 2% fetal bovine serum, 10 μg/ml cholesterol (C3045-5G; Sigma, Bengaluru, India), and 10 μM ATS (PZ0001-5MG; Sigma) for 24 h. For drug intervention studies, cells were pretreated with OICR-9429 (sml1209; Sigma) and resveratrol (R5010; Sigma) for 24 h. After 24 h of inhibitor treatment, cholesterol and ATS were added to the same plate. For RNA extraction, the cells were seeded in 24-well plates with a seeding density of 0.05 × 10^6^ per well.

### Immunoblotting

The liver harvested from HFD and HFD + statin-treated groups was homogenized using a hand homogenizer with RIPA lysis and extraction buffer (R0278; Sigma) and centrifuged at 12,000 rpm for 10 min to pellet the cell debris. The supernatant was collected in fresh tubes, and the protein concentration was quantified using a Pierce BCA protein Assay kit (Thermoscientific; 23225). Briefly, the lysate was mixed with 2× Laemmli buffer and incubated for 10 min at 100°C, and samples were resolved by SDS-PAGE and transferred to nitrocellulose membranes. The membranes were blocked using 5% skimmed milk and were incubated with the primary antibodies (supplemental Table S1) at 4°C overnight. The blots were washed three times with Tris-buffered saline with Tween-20 buffer and incubated with a secondary antibody. The antibody-antigen reactions were detected using the Clarity™ Western ECL Substrates (Bio-Rad, Kolkata, India). The blot images were taken using the ChemiDoc MP Imaging System (Bio-Rad). The optical density of the proteins was expressed as a ratio analysis of the optical density of GAPDH or total histone H3, detected in the same blot after stripping.

### Total RNA extraction, reverse transcription, and RT-PCR

Total RNA was extracted from the liver and cells using the TRIzol:chloroform:isopropanol method. Briefly, TRIzol was added to chopped liver tissue and homogenized using a hand homogenizer in microcentrifuge tubes incubated at room temperature for 5 min. To this, lysate chloroform was added to each sample and gently mixed by inverting the tubes and using vortex and incubated at room temperature for 5 min. The samples were then centrifuged at 12,000 *g* for 15 min at 4°C. A clear aqueous layer was formed at the top of the lysate mixture with sediment in the intersection. The upper aqueous layer was collected in a fresh centrifuge tube without disturbing the interphase. Isopropanol was added to the aqueous solution, mixed gently using the vortex mixer, and incubated at room temperature for 10 min. A white pellet was formed at the bottom of the tube. The supernatant was carefully discarded, and the pellet was washed twice with 75% ethanol, followed by centrifugation at 7,500 *g* at 4°C after each wash. The pellet was air-dried and resuspended in diethylpyrocarbonate-treated water. The purified RNA was quantified, and the quality was measured at an absorbance of 260:280 nm using Multiscan Go. About 2 μg of purified RNA was used for complementary DNA (cDNA) synthesis. cDNA was prepared using a High-Capacity cDNA Reverse Transcription Kit according to the manufacturer’s protocol (4368814; Invitrogen). The cDNA was subjected to real-time PCR quantification using SYBR Green Master Mix on an Applied Biosystems QuantStudio 5 Real-Time PCR System. Relative amounts of mRNAs were calculated using the comparative ΔΔCT method with 18s as endogenous control.

### Chromatin immunoprecipitation and PCR analyses

HepG2 cells were seeded (0.8 × 10^6^) in 60 mm culture dishes and treated with 10 μg/ml of cholesterol and 10 μM of ATS for 24 h. A chromatin immunoprecipitation (ChIP) assay was carried out according to the user manual provided by EpiQuik ChIP Kit (Farmingdale). In brief, the cells were washed with PBS following incubation in culture media containing 1% formaldehyde and incubated at room temperature for 10 min on a rocking platform (50–100 rpm). About 1.25 M glycine solution was added to the cells and following centrifugation at 1000 rpm for 5 min washed twice with ice-cold PBS. The cell pellet was resuspended using CP3A solution, incubated on ice for 10 min for cell lysis, and centrifuged at 5000 rpm for 5 min. The supernatant was collected, and CP3B solution containing protease inhibitor cocktail was added following incubation on ice for 10 min. DNA was fragmented using the micrococcal nuclear enzyme (NEB, Mumbai, India) digestion and incubated on ice for 20 min (fragment length: 200–1,000 bp) following the addition of EGTA to stop the activity of the enzyme. The fragmented DNA was immunoprecipitated using the ChIP antibody (supplemental Table S1). The samples were washed and reverse crosslinked to obtain purified DNA. The real-time PCR was performed using ChIP primers, and all the reactions were normalized using input DNA and presented as mean ± SD.

### Sample preparation and immunofluorescence

For histological processing, the animals were anesthetized using isoflurane, and the liver tissue was perfused using PBS solution to 50 ml/10 min. The liver was fixed in a 4% paraformaldehyde solution overnight. Postfixation, the liver was placed in 15% sucrose in PBS until the tissue sank and then transferred to 30% sucrose solution overnight. The tissue was embedded in OCT and frozen immediately using liquid nitrogen, and 7 μm thick sections were cut at a cryotome (Leica), and the section was stored at −20°C until use.

HepG2 cells were seeded in 24-well plates (seeding density = 0.1 × 10^6^ cells/well) and incubated at 37°C in a 5% CO_2_ incubator for 24 h. The cells were treated with respective treatments. Cells were fixed with 4% paraformaldehyde for 10 min at room temperature and washed with PBS thrice for 5 min. Cells or tissues were permeabilized with 0.1% Triton X-100 on ice for 5 min, following three 5 min washes with PBS, and subsequently blocked with 2% BSA for 1 h at room temperature. Further, the cells were incubated with primary antibodies (supplemental Table S1) overnight at 4°C. The cells were then stained with a secondary antibody for 1 h and counterstained with 4',6-diamidino-2-phenylindole (1 μg/ml) for 10 min. After three additional 5 min washes, fluorescence imaging was done using the Zeiss ApoTome 2.0 microscope (Carl Zeiss), and images were processed using ImageJ software (15).

### ELISA

PCSK9 serum level in the animals treated with HFD and HFD + STATIN was measured using a commercially available ELISA kit (Quantikine ELISA MPC900; R&D, Bengaluru, India). In brief, the blood collected from the animals was allowed to clot for 2 h at room temperature and then centrifuged for 20 min at 2,000 *g*. The serum was collected and stored at −80°C for further analysis. Briefly, serum and standards were added to the 96-well plate precoated with a mouse antibody against PCSK9 for 2 h at room temperature. After washing, mouse PCSK9 conjugate was added to the wells and incubated for 2 h at room temperature. In subsequent washing steps, substrate solution was added to the wells for enzymatic reaction followed by stop solution addition to the plates. The optical was determined using a microplate reader set to 450 nm.

### LDL uptake assay

HepG2 cells attached to coverslips were transfected with siPCSK9 or scrambled RNA using Lipofectamine (Lipofectamine™ RNAiMAX Transfection Reagent). After 24 h of transfection, cells were switched to DMEM supplemented with reduced serum-containing treatment groups, as mentioned earlier. About 48 h later, or 60–62 h post-transfection, cells were incubated with 10 μg/ml of fluorescently labeled LDL (DiI-labeled) for 3 h. The coverslips were washed twice with 3% BSA containing ice-cold PBS. After fixing with 4% paraformaldehyde, cells were counterstained with 4',6-diamidino-2-phenylindole, and the uptake of the LDL was identified using a Zeiss ApoTome 2.0 microscope.

### Statistics

Statistical significance was determined by Student’s *t*-test (a two-tailed distribution with a two-sample equal variance). A *P* value of less than 0.05 was considered statistically significant. All the values are expressed as the mean ± SD. Statistical differences for more than two groups were determined by a one-way ANOVA for comparisons between groups. Statistical analyses were performed using GraphPad Prism software (GraphPad Software, Inc).

## Results

### ATS induces the expression of PCSK9 by modulating histone methylation and acetylation marks

**Fig. 1:**
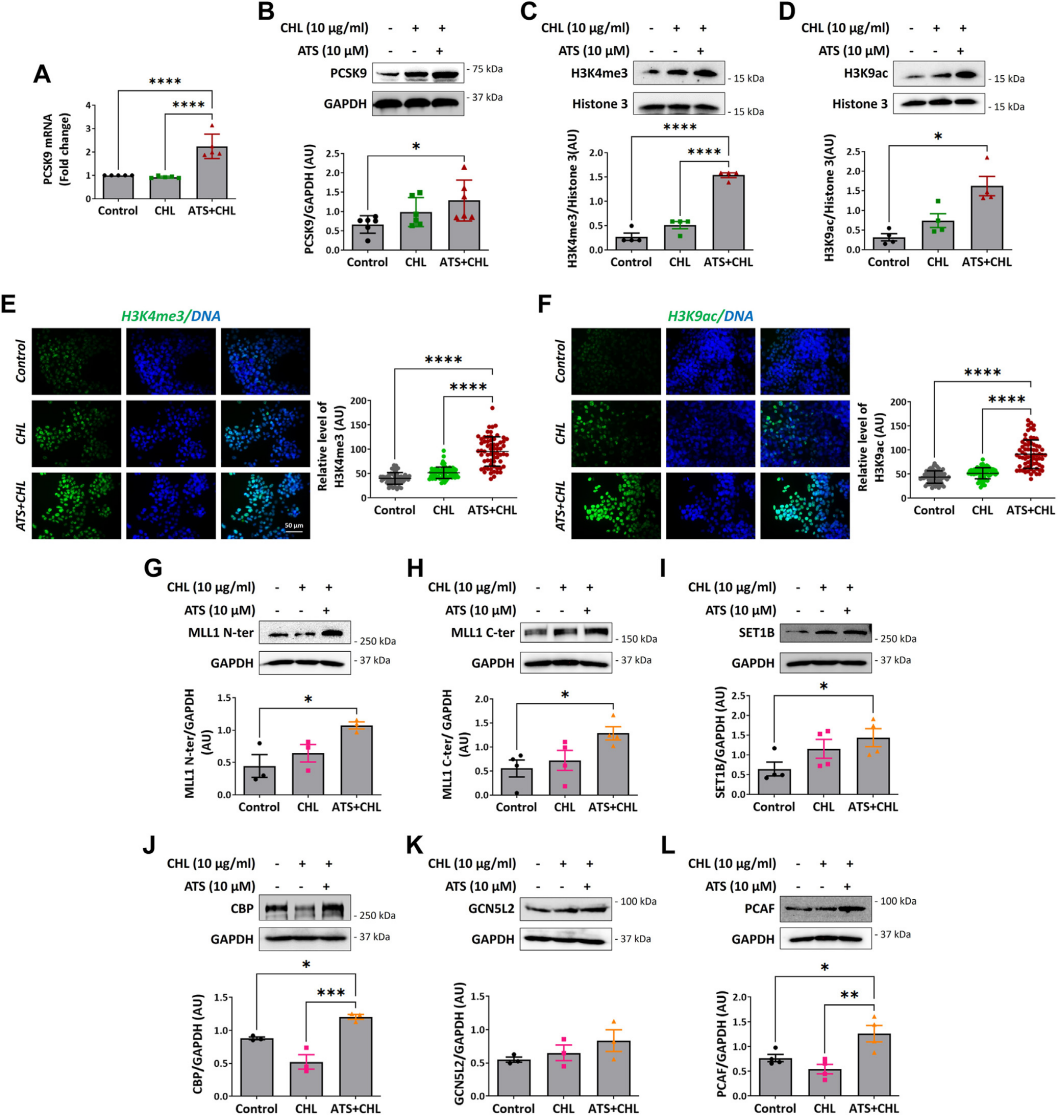
ATS elevates PCSK9 levels and enhances H3K4me3 and H3K9ac by inducing the expression of methyltransferases and acetyltransferases (A) (n = 4). *PCSK9* mRNA levels in HepG2 cells exposed to cholesterol (CHL; 10 μg/ml) with or without ATS (10 μM). C, D: immunoblotting for PCSK9 (n = 6) (B), H3K4me3 (n = 4) (C), and H3K9ac (n = 4) (D) in HepG2 cells treated with ATS (10 μM) and/or CHL (10 μg/ml). E, F: immunofluorescence staining for H3K4me3 (n = 4) (E) and H3K9ac (n = 4) (F) in HepG2 cells treated with ATS (10 μM) and/or CHL (10 μg/ml). 4',6-Diamidino-2-phenylindole staining is shown in blue. Fluorescence intensity (AU) values of individual cells from three independent experiments are indicated. Magnification: 40x, scale represents 50 μm. G–L: immunoblotting for MLL1 N-ter (n = 3) (G), MLL1 C-ter (n = 4) (H), SET1B (n = 4) (I), CBP (n = 3) (J), GCN5L2 (n = 3) (K), and PCAF (n = 4) (L) in HepG2 cells exposed to ATS (10 μM) with or without CHL (10 μg/ml). H3K4me3 and H3K9ac protein levels were normalized to total histone 3, and other protein levels were normalized to GAPDH. Values represent mean ± SD. ∗*P* < 0.05, ∗∗∗∗*P* < 0.0001, by one-way ANOVA.

**Supplemental Figure S1:**
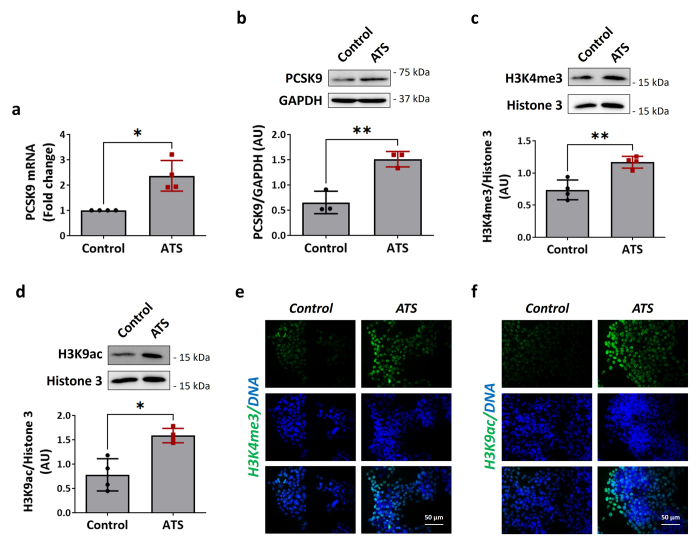


**Supplemental Figure S2:**
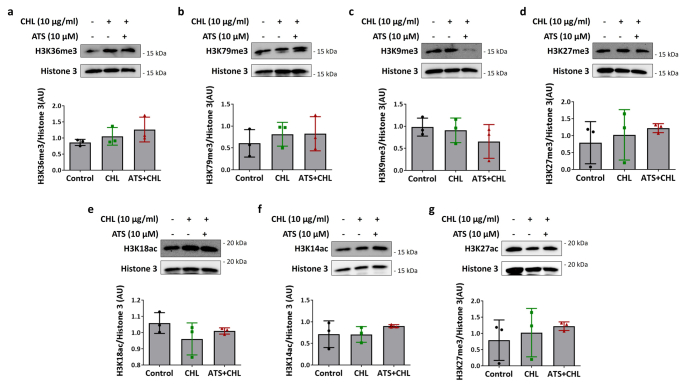


It is proven that statins increase the expression of PCSK9 by regulating the expression of SREBP2. In our study, first, we validated the effects of ATS on the regulation of PCSK9 expression. To mimic the in vivo disease condition, we treated HepG2 with ATS (10 μM) with or without high cholesterol (10 μg/ml). Our data revealed that the addition of ATS significantly upregulated both transcript and protein levels of PCSK9 under high cholesterol (Fig. 1A, B). In addition, we also showed that treatment with only ATS causes more than a 2-fold increase in the expression of PCSK9 (supplemental Fig. S1A, B). Statins are shown to induce post-translational modifications of histones (16), specifically histone 3; therefore, we began by assessing the enrichment levels of various histone H3 methylation and acetylation marks. In so doing, we evaluated the levels of five different lysine residue trimethylation marks in histone H3, namely H3K4me3, H3K9me3, H3K27me3, H3K36me3, and H3K79me3. Immunoblotting showed a 2-fold elevation in the levels of H3K4me3 in statin-treated cells (Fig. 1C). However, the other activating methyl marks like H3K36me3 and H3K79me3 remain unchanged (supplemental Fig. S2A, B). As the addition of methyl group may lead to either activation or repression of the gene, we also checked the modulation of repressive histone methylation marks like H3K9me3 and H3K27me3. Interestingly, we observed a slight decrease in the expression of H3K9me3 (supplemental Fig. S2C); however, the changes were not significant and the levels of H3K27me3 remained unchanged (supplemental Fig. S2D).

Next, following the screening of four different acetylation marks, H3K9ac, H3K14ac, H3K18ac, and H3K27ac, by immunoblotting, it showed elevation in the levels of H3K9ac in ATS and cholesterol-treated cells (Fig. 1D). The other acetylation marks like H3K18ac, H3K14ac, and H3K27ac remained unchanged (supplemental Fig. S2E–G). Interestingly, the marks that we observed to be significantly elevated, that is, H3K4me3 and H3K9ac, were activating histone marks, and enrichment of these marks to the promoter region leads to the transcriptional activation of genes. In addition, we also performed immunofluorescence that indicated the abundance of H3K4me3 and H3K9ac in statin-treated cells (Fig. 1E, F).

**Supplemental Figure S3:**
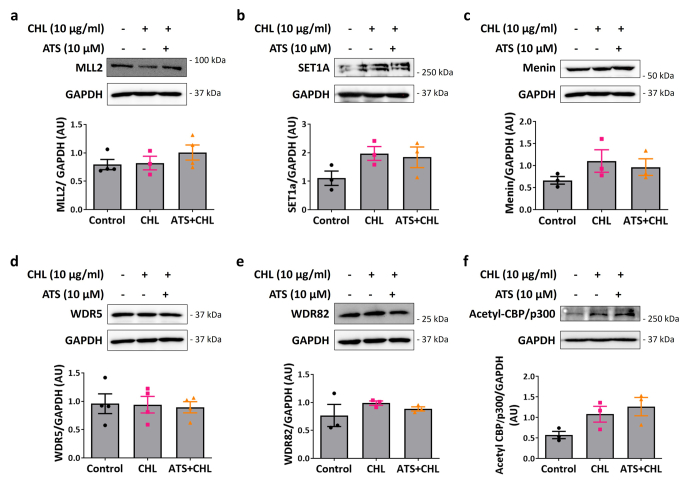


**Supplemental Figure S4:**
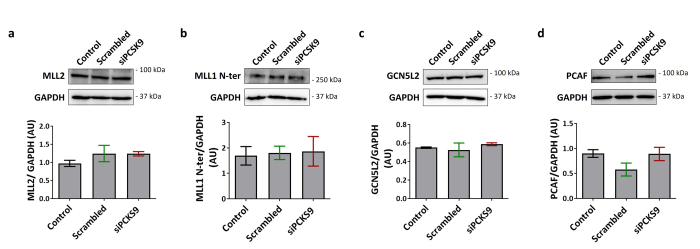


Since the addition of ATS elevates the levels of H3K4me3 and H3K9ac, we then examined the alteration in the activity of transferases specific to these histone modifications. SET1/COMPASS family of proteins adds a methyl group to the lysine 4 residue of histone 3. Immunoblotting showed upregulation of MLL1 N and C-terminal and SET1b (Fig. 1G–I) upon statin treatment; however, the other proteins like MLL2, SET1A, Menin, WDR5, and WDR82 remained unaltered (supplemental Fig. S3A–E). This confirms that the activation of H3K4me3 upon statin treatment is driven by the MLL1 and SET1 complex of proteins. Similarly, we then investigated the changes in the acetyl transferases complex like CBP, PCAF, and GCN5L2, which are known acetyltransferases. Upon statin treatment, we observed significant upregulation in the expression of CBP and PCAF protein levels (Fig. 1J, L). These findings interest us in further exploring whether modulating the expression of PCSK9 would affect the regulation of methyl and acetyltransferases. Silencing the expression of *PCSK9* in HepG2 revealed no significant effect on the expression of methyltransferase proteins like MLL1,2 and acetyltransferases like PCAF and GCN5L2 (supplemental Fig. S4).

### ATS increases the fold enrichment of activating histone marks to the promoter region of *Pcsk9*

**Fig. 2:**
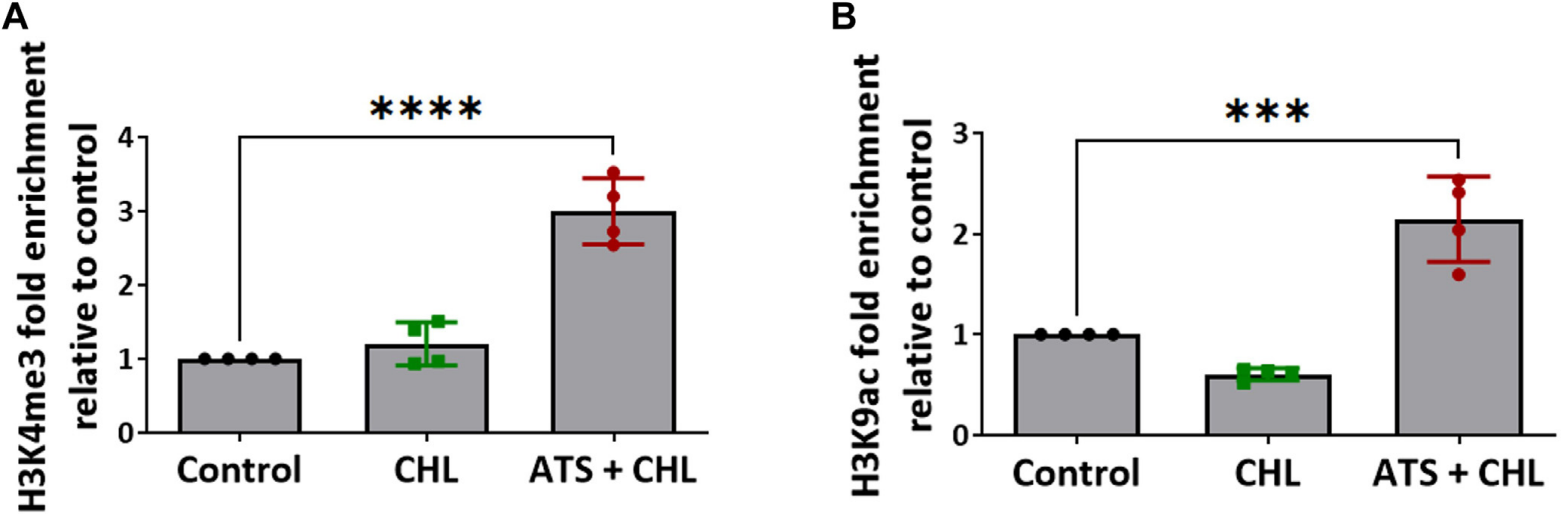
ATS causes H3K4me3 and H3K9ac enrichment at the *PCSK9* gene promoter (A, B) Chip-qPCR for *PCSK9* gene promoter in the DNA of HepG2 cells treated with cholesterol (10 μg/ml) and ATS (10 μM) immunoprecipitated with H3K4me3 (n = 4) (A) and H3K9ac (n = 4) (B) antibodies. Input samples were used as internal control and represent total chromatin. Data were expressed as fold change of ATS and cholesterol-treated cells with the expression of control samples. Values represent mean ± SD. Data were analyzed using one-way ANOVA. ∗∗*P* < 0.01, ∗∗∗*P* < 0.001.

Once we ascertained a significant upregulation in the expression of PCSK9 upon statin treatment, we next examined whether activation of PCSK9 expression was associated with changes in the histone H3K4me3 and H3K9ac marks. We performed ChIP-quantitative PCR (qPCR) to confirm the enrichment of H3K4me3 and H3K9ac to the promoter region of *PCSK9* under ATS treatment in HepG2. ChIP assay demonstrated more than 2-fold enrichment of both H3K4me3 and H3K9ac at the gene promoter region of *PCSK9* (Fig. 2A, B). These findings confirm the elevation of the methylation and acetylation marks upon statin treatment leads to an increase in the enrichment of K4me3 and K9ac to the promoter of *PCSK9*. Furthermore, our data reveal the colocalization of H3K4me3 and H3K9ac to the promoter region of *PCSK9*, which further recruits the transcription machinery for active gene expression.

### ATS administration in HFD-fed mice increases H3K4me3, H3K9ac, and subsequently PCSK9 expression

**Fig. 3:**
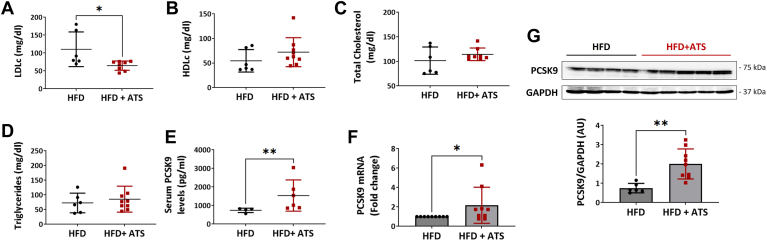
In vivo ATS administration with HFD increases the serum and liver PCSK9 expression (A–D) serum LDL-C (A), HDL-C (B), total CHL (C), and triglycerides (D) from HFD (n = 6) and HFD + ATS (n = 9) (10 mg/kg) fed mice (E) ELISA for PCSK9 expression in the serum of mice from HFD and HFD + ATS (10 mg/kg) fed groups. F: *Pcsk9* mRNA levels in the liver of HFD and HFD + ATS (10 mg/kg) fed mice groups. G: immunoblotting for PCSK9 in the liver of mice fed with HFD and HFD + ATS (10 mg/kg). Values represent mean ± SD. ∗*P* < 0.05, ∗∗*P* < 0.01, by unpaired *t*-test.

**Fig. 4:**
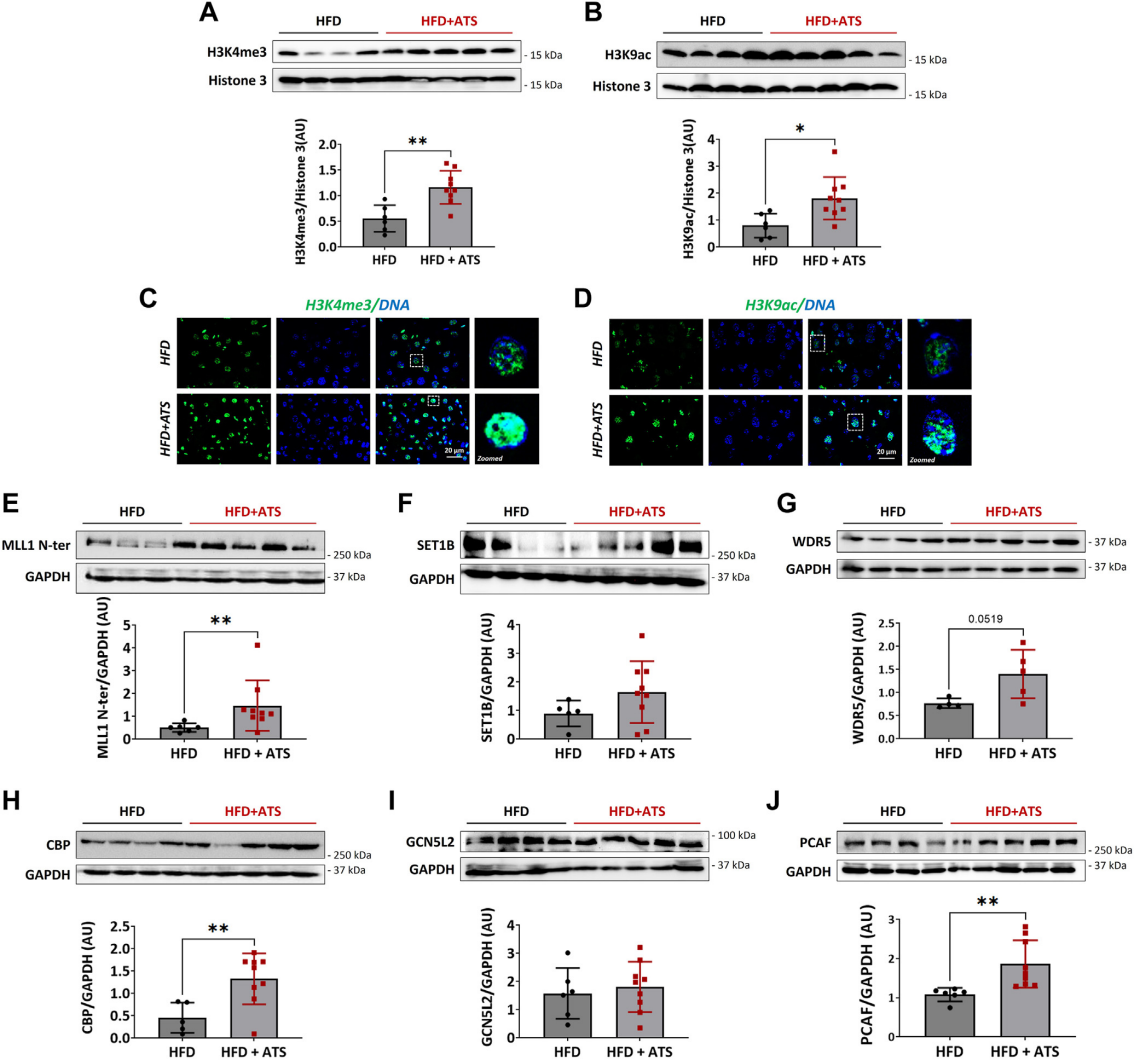
ATS enriches MLL1 and CBP/PCAF-driven H3K4me3 and H3K9ac in HFD-fed mice. Whole liver sections and extracted proteins were analyzed in HFD (n = 6) and HFD + ATS (n = 9). A, B: immunoblotting for H3K4me3 and H3K9ac; immunofluorescence of H3K4me3 (C) and H3K9ac (D). Cells were stained with 4',6-diamidino-2-phenylindole (blue), and merge images show colocalization of H3K4me3 or H3K9ac with 4',6-diamidino-2-phenylindole. Magnification 60X, scale represents 20 μm. Immunoblotting for MLL-1n (E), SET1B (F), WDR5 (G), CBP (H), GCNL52 (I), and (J) PCAF in the treated and untreated mice. H3K4me3 and H3K9ac protein levels were normalized to total histone 3, and other protein levels were normalized to GAPDH. Values represent mean ± SD. Data were analyzed using unpaired *t*-tests for treatment versus HFD comparisons. ∗*P* < 0.05, ∗∗*P* < 0.01.

**Supplemental Figure S5:**
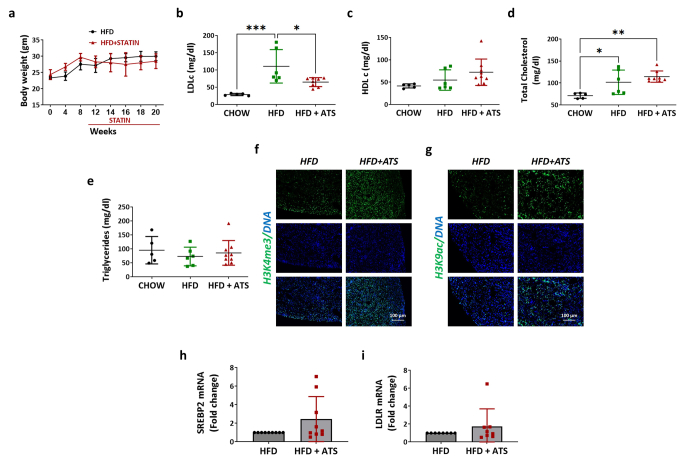


**Supplemental Figure S6:**
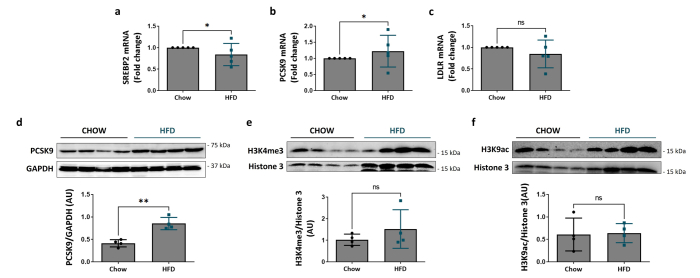


In vitro results showed that treatment with high cholesterol and ATS in hepatocytes elevated the H3K4me3 and H3K9ac marks. Similarly, we investigated the change in the regulation of histone marks using an animal model. To this end, we divided WT mice into two groups; both groups of mice were fed HFD for 20 weeks. After 10 weeks of HFD, one of the two groups was administered ATS (10 mg/kg) via drinking water for the remaining 10 weeks. Observations revealed no change in the body weights of the animals in either group (supplemental Fig. S5A). The serum LDL-C levels were significantly lower in the statin group than in the HFD group. However, serum HDL-C levels marginally increased in the statin group. No change was observed in the triglyceride and total cholesterol levels between the groups (Fig. 3A–D). First, we checked the PCSK9 levels in the HFD group compared to the chow animals. Interestingly, 20-week-fed HFD animals showed a significant increase in levels of PCSK9 at both transcript and protein levels (supplemental Fig. S6B, D). We then assessed the *SREBP-2* and *LDLR* mRNA levels and observed reduced *SREBP-2* (supplemental Fig. S6A) and unchanged *LDLR* (supplemental Fig. S6C) transcripts. In addition, we also checked the levels of methylation and acetylation marks in the HFD-fed mice; the results revealed that modest to no significant changes were observed in H3K4me3 and H3K9ac (supplemental Fig. S6E, F) levels. As our study focuses on the regulation of statin-mediated epigenetic changes, which closely reflect clinical changes, we continued our study with HFD and HFD + ATS groups of mice. Our data revealed a significant increase in the serum PCSK9 levels in the statin-treated HFD-fed group (Fig. 3E). In addition, we also observed an increase in the liver transcript and protein levels of PCSK9 in statin-treated HFD-fed mice (Fig. 3F, G). Data also showed increased transcript levels of *SREBP2* and *LDLR* in the statin group (supplemental Fig. S5H, I). Furthermore, immunoblotting analysis revealed that ATS significantly elevated the levels of H3K4me3 and H3K9ac in the liver of HFD with the statin group (Fig. 4A, B). In addition, immunofluorescence-based histological analysis confirms increased levels of H3K4me3 and H3K9ac in the liver of the two groups (Fig. 4C, D). We also assessed the protein level expression of histone methyltransferase in the liver of statin-treated HFD-fed mice. Immunoblotting results showed a significant increase in the MLL-1 expression and WDR5 (Fig. 4E, G); however, the levels of the other proteins like SET1b remain unchanged (Fig. 4F). We also evaluated the expression of acetyltransferases, which showed a significant increase in the CBP and PCAF (Fig. 4H, J) protein levels, but other proteins like GCN5L2 remain unchanged (Fig. 4I).

### Pharmacological inhibitors against H3K4me and H3K9ac reverse the statin-induced expression of PCSK9

**Fig. 5:**
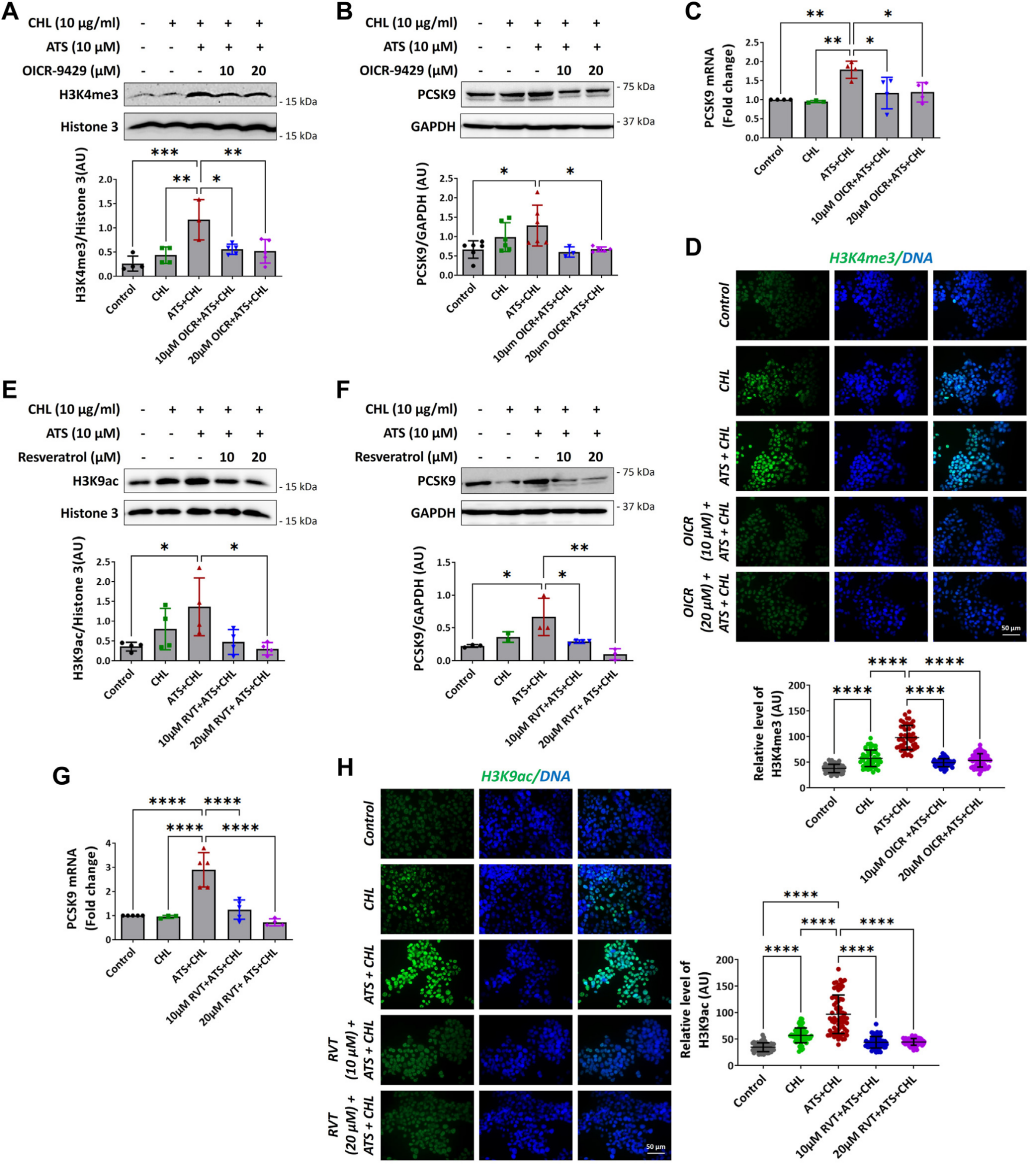
Pharmacological inhibition of the activating histone marks reversed the ATS-induced upregulation of PCSK9 immunoblotting for H3K4me3 (n ≥ 4) (A) and PCSK9 (n ≥ 5) (B) in HepG2 pretreated with MLL inhibitor, OICR-9429 (10 μM and 20 μM, 18 h) in combination with ATS (10 μM), with or without cholesterol (10 μg/ml, 24 h). C: *PCSK9* mRNA (n = 4) levels in the cells pretreated OICR-9429 and ATS with or without cholesterol with 18s as endogenous control. D: immunofluorescence of H3K4me3 (n = 4) in cell nuclei of HepG2 and the fluorescence intensity AU values were calculated per individual cells and are indicated together with mean and SD. Immunoblotting for H3K9ac (n = 4) (E) and PCSK9 (n = 4) (F) in HepG2 pretreated with RVT (10 μM and 20 μM, 18 h) in combination with ATS (10 μM) with or without cholesterol (10 μg/ml, 24 H). G: *PCSK9* mRNA (n ≥ 4) levels in the cells pretreated with RVT and ATS with or without cholesterol (H) H3K9ac (n = 4) expression in HepG2, the fluorescence intensity AU were calculated per individual cell and computed as mean and SD. H3K4me3 and H3K9ac protein levels were normalized to total histone H3 protein levels, and PCSK9 protein levels were normalized to GAPDH levels. Values represent mean ± SD. Data were analyzed using one-way ANOVA for treatment versus control comparisons. ∗*P* < 0.05, ∗∗*P* < 0.01, ∗∗∗∗*P* < 0.0001. RVT, resveratrol.

We speculated whether blocking the methylation of H3K4 would reverse the PCSK9 expression. To determine this, we pretreated cells with OICR-9429 (10 μM and 20 μM), a small-molecule inhibitor (which disrupts WDR-MLL interaction to block the catalysis of H3K4me3), followed by ATS and cholesterol. Immunoblotting analysis revealed a reduction in the levels of H3K4me3 in hepatocytes (Fig. 5A). We also confirmed the effects of OICR-9429 using immunofluorescence, which showed a significant decrease in the H3K4me3 (Fig. 5D). Correspondingly, we also determined the expression of PCSK9 upon treatment with OICR-9429. Through such assessment, we observed a significant reduction in the expression of PCSK9 at both protein and transcript levels (Fig. 5B, C). This confirmed that statin regulates the expression of PCSK9 via MLL-mediated elevation of H3K4me3 levels, and inhibiting the histone methyl transferase activity reverses the statin-induced upregulation of PCSK9.

Our in vitro and in vivo data showed that the administration of statin significantly elevated the acetylation of H3K9. Along similar lines, we asked whether the reversal of histone acetylation by targeting histone deacetylase (HDAC) would reverse the effects of statin. As the literature suggests epigenetic regulation of PCSK9 by SIRT6, we used resveratrol (10 μM and 20 μM), which is a polyphenol compound known to activate SIRT6, an NAD^+^-dependent HDAC (17). First, we confirmed the effects of resveratrol on *SIRT6* by qPCR, and we observed a 2-fold increase in the expression of *SIRT6* (supplemental Fig. S6). Second, upon pretreatment of cells with resveratrol followed by treatment with statin and cholesterol, we observed a significant decrease in the levels of H3K9ac. In addition, immunofluorescence staining confirmed the benefits of resveratrol (Fig. 5E, H). Further, we checked the direct effects of resveratrol on the regulation of PCSK9. Our data revealed resveratrol reverses the statin-dependent upregulation of PCSK9 (Fig. 5F, G). So, this concludes that resveratrol reduces the acetylation levels by activating HDACs and reverses the statin-induced upregulation of PCSK9.

### Combination of statin and OICR-9429 or resveratrol increases the LDL uptake in hepatocytes

**Fig. 6:**
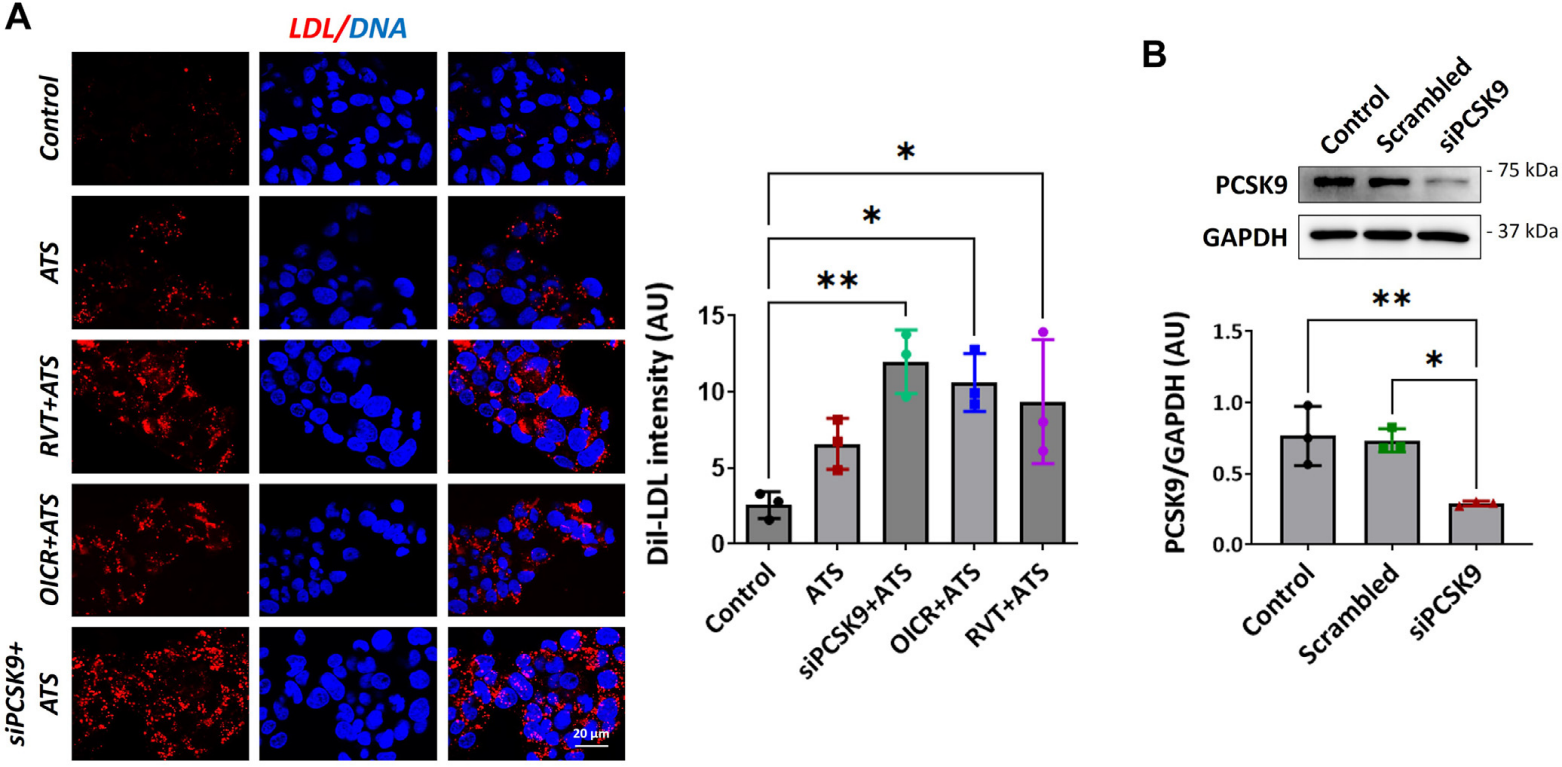
Inhibition of PCSK9 in combination with ATS improved the LDL uptake by hepatocytes Representative images are shown for the fluorescent LDL uptake after 3 h of incubation. A: the images of fluorescent dye-labeled LDL endocytosed by the cells (shown in red) and the nuclei (blue) were merged to show LDL uptake by each cell. The LDL influx by cells was calculated by weighted mean Dil-LDL intensities and nuclei count for each cell population. Values represent mean ± SD, and data points are the average weighted mean of three independent experiments. Cells (n ≥ 50) from at least 10 different microscopic frames were analyzed for each independent experiment (n = 3). B: the representative blots for PCSK9 and GAPDH show reduced protein levels normalized to GAPDH. Data were analyzed using one-way ANOVA for treatment versus control comparisons. ∗*P* < 0.05, ∗∗*P* < 0.01. RVT, resveratrol.

Treatment with OICR-9429 or resveratrol reversed the statin-induced upregulation of PCSK9. Furthermore, this interests us to check whether these inhibitors, along with statins, would improve the overall LDL uptake by cells by LDLR, a functional examination for their suitability in reducing the free LDL. First, we treated cells with inhibitors in the absence of high cholesterol, followed by the addition of ATS (10 μM) for 24 h. Last, DiI-LDL was added for 3 h before fixing the cells with formaldehyde. The weighted mean intensity revealed a significant increase in the uptake of LDL in OICR-9429 and resveratrol-treated groups compared to the only ATS groups. As a proof of concept, a positive control group, where PCSK9 expression was silenced using siRNA (Fig. 6B), followed by treatment with ATS. The DiI-LDL uptake in the PCSK9 silenced group was drastically increased compared to only the statin-treated cells (Fig. 6A). An increase in the uptake of DiI-LDL by the cells in the PCSK9-inhibited group suggests elevation in the number of LDLR expressed on the membrane of hepatocytes. This confirms that inhibition of H3K4me3 and H3K9ac lowers PCSK9 levels and restores the LDLR to the surface of hepatocytes, which helps improve LDL uptake.

## Discussion

In this study, we demonstrated that statin regulates the expression of PCSK9 by modulating the levels of H3K4me3 and H3K9ac in hepatocytes. ATS elevates the expression of methyl transferases and acetyltransferases, which facilitates the enrichment of H3K4me3 and H3K9ac in the *PCSK9* gene promoter. Notably, colocalization of H3K4me and H3K9ac leads to the activation of the PCSK9 gene transcription. Mechanistically, inhibiting these activating histone marks using OICR-9429 or resveratrol reversed the statin-induced upregulation of PCSK9. A combination of statins and pharmacological inhibitors significantly improved the LDL uptake in hepatocytes. Thus, we showed the pleiotropic effects of statins at multiple levels, from modulating histone marks to activating methyl and acetyltransferase activity. These findings broaden our understanding of statin-mediated PCSK9 regulation and suggest a possible reason behind statin resistance that limits patient utility.

Statins inhibit the cholesterol de novo synthesis that causes the activation of SREBP-2, further regulating the expression of LDLR (11). An increase in the surface LDLRs on hepatocytes helps in the clearance of LDL-C from serum. Thus, statins are the most prescribed lipid-lowering drugs for treating hypercholesterolemia (2). However, activation of SREBP-2 also increases the activity of PCSK9, while statins effectively decrease cholesterol levels; their efficacy is diminished by this increase in PCSK9 (10, 18). A recent study on statin naïve coronary artery disease patients showed a relationship between circulating mature PCSK9 and statin hyporesponsiveness in these patients. Post-a-month treatment with statin, 11% of the patients showed baseline mature PCSK9 levels >228 ng/ml associated with hyporesponsiveness to statin (19). Similar findings suggest that doubling the statin dosage (80 mg) caused a rapid increase in serum PCSK9 levels by 47% within 4 weeks of treatment (20). Furthermore, a randomized clinical trial treatment of patients with simvastatin, ezetimibe, and a combination of both revealed only simvastatin led to an increase in serum PCSK9 levels. However, only ezetimibe or the combination of both drugs is not associated with the increase in PCSK9 levels (21). These findings suggest a strong correlation between statins and PCSK9 levels in patients; thus, it is critical to understand the different ways statins modulate the expression of genes. Statins have been shown to alter gene expression by inhibiting HDAC activity and influencing many miRNAs in both in vitro and animal models (22). Previously, studies have proposed that inhibition of cholesterol biosynthesis diverted the use of acetyl CoA by HATs to elevate the histone acetylation; however, this has not been proven yet (23). Tao *et al.* revealed that PCSK9 can also be regulated by epigenetic marks like acetylation of H3K9 and H3K56 (13, 14). These indicate that PCSK9 can also be regulated by epigenetic modification, and statins are known for its pleiotropic effects; therefore, we investigated the statin-mediated epigenetic regulation of PCSK9 expression. Initially, we checked the PCSK9 levels in 20-week HFD-fed mice and observed a 2-fold increase in the expression of hepatic PCSK9. Similar observations were reported in other studies, where administration of HFD for more than 8 weeks significantly increased the hepatic PCSK9 levels by more than 2-fold (13, 24). In contrast, the hepatic PCSK9 was reduced when the HFD was fed only for 24 h or 1 week (25, 26). This shows that chronic exposure to HFD leads to high PCSK9 levels. We also investigated the methylation and acetylation levels in the HFD-fed mice, and these modifications showed limited changes. However, our data revealed that ATS administration, and high cholesterol, significantly induces the levels of activating histone marks H3K4me3 and H3K9ac in both HepG2 and statin-fed mice groups. In addition, we also showed that treatment of hepatocytes only with ATS leads to a significant increase in the levels of H3K4me3 and H3K9ac (supplemental Fig. S1C–F). Previously, it was reported that colocalization of H3K4me3 and H3K9ac leads to active gene transcription. Gates *et al.* (27) demonstrated that H3K4me3 promotes transcription initiation, and H3K9ac is involved in the elongation of the gene by releasing the pol II pause and recruiting the superelongation complex to the chromatin. Our ChIP-qPCR results showed the enrichment of activating marks H3K4me3 and H3K9ac to the promoter, suggesting statins involvement in the active gene transcription of *Pcsk9*.

To date, our understanding of how statins modulate histone methylation is very limited. However, a recent study illustrated that HFD-fed *Caenorhabditis elegans* showed elevated H3K4me3, which further induced transcription factors like SREBP, DAF-16/FOXO, and nuclear receptors NHR-49 and NHR-80. Among them, only SREBP responded to alteration in H3K4me3 and activated genes involved in lipid metabolism leading to multigenerational obesogenic effects (28). This shows that H3K4me3 plays a crucial role in cholesterol metabolism. Since we reported that statin increases the levels of H3K4me3, we were interested in looking for the expression of the methyl transferases and their binding partners/associated proteins. The SET1 and MLL1 proteins of the SET1/COMPASS contain the catalytic domain responsible for the monomethylation, dimethylation, and trimethylation of H3K4 (29). We checked the expression of the SET1/COMPASS family of proteins and observed an increase in the expression of SET1b and MLL1-n in the statin-treated group of hepatocytes and liver of statin-treated mice. This study for the first time reported statin-mediated elevation of H3K4me3, which further increases PCSK9. In addition to this, we also assessed the alteration in the expression of HATs under statin treatment. HATs like p300, CBP, GCNL5, and PCAF are transcriptional coactivators, and localization of these to the promoter causes active gene transcription. Deletion of GCN5/PCAF in mouse embryonic fibroblasts drastically reduces the acetylation of H3K9 (30). Thus, we evaluated the HAT expression, and our data revealed an increase in the expression of CBP and PCAF in the liver of the statin-treated group of animals. This suggests that statin increases the HAT expression, further elevating H3K9ac. Similarly, a recent study emphasizes that p300 acetyltransferase inhibition using piceatannol reverses the statin-mediated upregulation of PCSK9 (14). All these findings suggest that increased serum PCSK9 is a result of alterations of histone marks by statins, which further leads to resistance and toxicity.

To further corroborate these results, we inhibited the alteration of histone marks by using pharmacological inhibitors. One of the well-known small-molecule inhibitors of H3K4me3 is OICR-9429 (31). OICR-9429 treatment in hepatocytes reduced the methylation of H3K4 and reversed the statin-induced upregulation of PCSK9. As studies have shown, SIRT6 is one of the regulators of PCSK9. We treated cells with resveratrol, which is involved in the direct activation of sirtuins (32, 33). Similarly, we observed a significant increase in the expression of *SIRT6* in the presence of resveratrol. Upon treating cells with resveratrol, we observed a significant reduction in the levels of H3K9ac and inhibited the statin-mediated upregulation of PCSK9. As these epigenetic inhibitors successfully decreased the expression of PCSK9, we further checked the overall LDL uptake of hepatocytes. The presence of both inhibitors and statin recuperated the uptake of LDL by hepatocytes. This indicates that the administration of histone methyl transferase or acetyltransferase inhibitors along with statin may help reduce statin resistance and enhance the clearance of LDL-C from the serum.

The epigenetic changes are known to be controlled by many external factors, which consecutively regulate the expression of genes in the progression of several diseases (31, 34, 35, 36). Post-translational modification of histone methylation and acetylation has recently been acknowledged for their involvement in the progression and development of atherosclerotic plaque by regulating the epigenome modification in different cells (34). Moreover, lately, a methyl transferase inhibitor called tazemetostat has been Food and Drug Administration approved to stop cancer cell dedifferentiation. These findings shed light on epigenetic modifiers as the crucial regulators of various diseases. Similarly, a comprehensive understanding of the epigenetic regulation of PCSK9 will help design potential therapeutic strategies to counter the adverse effects of statins in hyperlipidemia patients.

Together, these findings suggest that statins modulate the expression of PCSK9 by regulating the enrichment levels of H3K4me3 and H3K9ac at the PCSK9 gene promoter. Inhibition of these activating histone modifications repressed statin-dependent PCSK9 expression. Although we reported inhibiting PCSK9 with OICR-9429 or resveratrol improves the LDL uptake in hepatocytes, the lack of pharmacological intervention in animal models limits our understanding of how these inhibitors will help reduce the serum LDL-C and overcome statin resistance. In hepatocytes, statin treatment increases the expression of H3K4me3 by upregulating the expression of SET1B and MLL1; however, specific knockdown of SET1B or MLL1 proteins will provide interesting insights into the involvement of the methyltransferase with PCSK9 regulation. Additional studies are required to investigate the underlying mechanisms involved in statin-mediated epigenomic elevation of PCSK9 in an in vivo setting. In addition, mapping the statin-mediated alterations in the epigenome that regulate PCSK9 expression and other key molecular players would provide important insights into its physiological implications. Despite the above limitations, our study strongly suggests the regulation of PCSK9 by statins via epigenetic pathways. Thus, chromatin remodeling may help negate the adverse effects of statin treatment, primarily of increasing PCSK9 during hypercholesterolemia.

## Data availability

The data that support the findings of this study are available from the corresponding author upon reasonable request.

## Supplemental data

This article contains supplemental data.

## Ethics approval

Approved by the Institutional Animal Ethics Committee, IIT Kharagpur (protocol no: IE-1/PCS-SMST/2.19).

## Conflict of interest

The authors declare that they have no conflicts of interest with the contents of this article.

## Figures and Tables

**Supplemental Figure S7:**