# Supplementary material for: New epigenome players in the regulation of PCSK9-H3K4me3 and H3K9ac alterations by statin in hypercholesterolemia
Source: J Lipid Res. 2024 Nov 19;66(1):100699. doi: 10.1016/j.jlr.2024.100699 (PMC11699316; doi:10.1016/j.jlr.2024.100699)
Supplement: Supplemental Figure Legends [file mmc1.docx]

**New Epigenome Players in the Regulation of PCSK9 – H3K4me3 and H3K9ac Alteration by Statins in hypercholesterolemia**

Sushmitha Duddu^1^, Yash T. Katakia^3^, Rituparna Chakrabarti^1^, Pooja Sharma^1^, Praphulla Chandra Shukla^1, 2, *^

1. School of Medical Science and Technology, Indian Institute of Technology Kharagpur, West Bengal, India
2. Human Medicine, Carl von Ossietzky University, Oldenburg 26129, Germany
3. Department of Biological Sciences, Birla Institute of Technology and Science (BITS), Pilani Campus, Pilani, India

*Correspondence:

Praphulla Chandra Shukla, PhD

School of Medical Science and Technology (SMST),

Indian Institute of Technology (IIT) Kharagpur,

Kharagpur 721302, West Bengal, India.

E-mail: [pcshukla@smst.iitkgp.ac.in](mailto:pcshukla@smst.iitkgp.ac.in), praphulla.shukla@uol.de

**Supplemental Fig S1** **Effect of atorvastatin on PCSK9, histone H3 methylation and acetylation marks** HepG2 cells were treated with atorvastatin (10 µM) for 24 h. *Pcsk9* mRNA (n=4)(a), Immunoblotting for PCSK9 (n=4)(b), H3K4me3 (n=4)(c), H3K9ac (n=4)(d), Immunofluorescence staining for H3K4me3 (n=4)(e) and H3K9ac (n=4)(f) in HepG2 cells treated with ATS (10 µM). DAPI staining is shown in blue. Magnification: 40x, Scale: 50 µm. Values represent mean ± SD. Data were analysed using unpaired t-test for treatment Vs. HFD comparisons. *= p< 0.05, **= p<0.01 Vs. control.

**Supplemental Fig S2** **Effect of atorvastatin and cholesterol on histone H3 methylation and acetylation marks** HepG2 cells were treated with atorvastatin (10 µM) with or without cholesterol (10 µg/ml) for 24h. Immunoblotting for H3K36me3 (n=3)(a), H3K79me3 (n=4)(b), H3K9me3 (n=4)(c), H3K27me3 (n=4)(d), H3K18ac (n=4)(e) H3K14ac (n=4)(f), and H3K27ac(n=4)(g). Above mentioned protein levels were normalized to total histone H3 protein levels. Values represents mean ± SD. Data were analysed using one-way ANOVA. CHL- cholesterol, ATS- atorvastatin

**Supplemental Fig S3 Effect of atorvastatin and cholesterol on SET/COMPAS family of proteins and histone acetyltransferases** HepG2 cells were treated with atorvastatin (10 µM) with or without cholesterol (10 µg/ml) for 24 h. Immunoblotting for MLL 2 (n=4)(a) SET1a (n=4)(b) and Menin (n=3) (c) WDR5 (n=4)(d) WDR 82 (n=3)(e) and acetyl CBP (n=4)(f). Above mentioned protein levels were normalized to total GAPDH protein levels. Values represents mean ± SD. Data were analysed using one-way ANOVA. CHL- cholesterol, ATS- atorvastatin

**Supplemental Fig S4 Changes in SET/COMPAS family of proteins and histone acetyltransferases post silencing the expression of *Pcsk9*** Immunoblotting for MLL2 (n=3)(a), MLL1- N-ter (n=3)(b), GCN5L2 (n=3)(c) and PCAF (n=3)(d) in HepG2 post silencing the PCSK9 expression. Above mentioned protein levels were normalized to total GAPDH protein levels. Values represent mean ± SD. Data were analysed using one-way ANOVA.

**Supplemental Fig S5** ***In vivo* atorvastatin administration effects on the Blood parameters and transcript level expression of SREBP2 and LDLR** Body weights (gm) of mice from HFD and HFD+ATS (10 mg/kg) fed groups (a). (b-e) Serum LDL-c (b), HDL-c (c), Total CHL (d), and Triglycerides (e) from Chow (n=5), HFD (n=6) and HFD+ATS (n=9) (10 mg/kg) fed mice. Immunofluorescence staining for H3K4me3 (n=3) (f) and H3K9ac (n=4) (g) HFD and HFD+ATS (10 mg/kg) fed mice. DAPI staining is shown in blue. Magnification: 10x, Scale: 100 µm. SREBP2 (h) and LDLR (i) mRNA levels in HFD + atorvastatin (10mg/kg) treated mice compared to HFD group. Values represent mean ± SD. Data were analysed using unpaired t-test for treatment Vs. HFD comparisons and for more than two groups one-way ANOVA was used. *= p< 0.05, **= p<0.01 Vs. control. HFD- High fat diet

**Supplemental Fig S6 Resveratrol increases the *Sirt6* expression in atorvastatin exposed hepatocytes** *Sirt6* mRNA (n=4) levels in cells pre-treated with resveratrol (10 µM 18 h) in combination with atorvastatin (10 µM) with or without cholesterol (10 µg/ml, 24 h). Each column represents mean ± SD. Data were analysed using one-way ANOVA. CHL- cholesterol, ATS- atorvastatin, RVT- resveratrol
