# Supplementary material for: New epigenome players in the regulation of PCSK9-H3K4me3 and H3K9ac alterations by statin in hypercholesterolemia
Source: J Lipid Res. 2024 Nov 19;66(1):100699. doi: 10.1016/j.jlr.2024.100699 (PMC11699316; doi:10.1016/j.jlr.2024.100699)
Supplement: Supplemental Table S1 [file mmc2.docx]

**Supplemental Table 1.** List of antibodies

| **Antibodies for Immunoblotting** | | |
| --- | --- | --- |
| Rabbit mAb H3K9ac | Cell Signaling Technology (Danvers, USA) | Cat#9649 |
| Rabbit mAb H3K14ac |  | Cat#7627 |
| Rabbit mAb H3K18ac |  | Cat#13998 |
| Rabbit mAb H3K27ac |  | Cat#8173 |
| Rabbit mAb H3K4me3 |  | Cat#9751 |
| Rabbit mAb H3K9me3 |  | Cat#13969 |
| Rabbit mAb H3K27me3 |  | Cat#9733 |
| Rabbit mAb H3K36me3 |  | Cat#4909 |
| Rabbit mAb H3K79me3 |  | Cat#4260 |
| Rabbit mAb Histone 3XP |  | Cat#4499 |
| Rabbit mAb SET1A |  | Cat#61702 |
| Rabbit mAb SET1B |  | Cat#44922 |
| Rabbit mAb MLL1 (Amino-termianl) |  | Cat#14689 |
| Rabbit mAb MLL1 (Carboxy-terminal) |  | Cat#14197 |
| Rabbit mAb MLL2 |  | Cat#63735 |
| Rabbit mAb WDR5 |  | Cat#13105 |
| Rabbit mAb WDR82 |  | Cat#99715 |
| Rabbit mAb Menin |  | Cat#6891 |
| Rabbit mAb CBP |  | Cat#7389 |
| Rabbit mAb Acetyl CBP |  | Cat#4771 |
| Rabbit mAb PCAF |  | Cat#3378 |
| Rabbit mAb GCN5L2 |  | Cat#3305 |
| Anti-Rabbit IgG, HRP-linked |  | Cat# 7074 |
| Rabbit pAb PCSK9 | Invitrogen, Mumbai, India | Cat#PA5-96836 |
| Rabbit pAb GAPDH | Merck, Bengaluru, India | Cat#ABS16 |
| **Antibodies for ChIP assay** | | |
| Rabbit polyclonal Ab H3K9ac ChIP-grade | Abcam (Singapore) | Cat#ab10812 |
| Rabbit polyclonal Ab H3K4me3 ChIP-grade |  | Cat#ab8580 |
| **Antibodies for Immunofluorescence** | | |
| Goat anti-Rabbit IgG Cross-Adsorbed Secondary Ab Alexa Fluor^TM^ 488 | Invitrogen | Cat#A11008 |
